# Supplementary material for: Transcriptomic Characterization of Cow, Donkey and Goat Milk Extracellular Vesicles Reveals Their Anti-inflammatory and Immunomodulatory Potential
Source: Int J Mol Sci. 2021 Nov 25;22(23):12759. doi: 10.3390/ijms222312759 (PMC8657891; doi:10.3390/ijms222312759)
Supplement: Supplementary file 1 [file ijms-22-12759-s001.zip › Supplementary_files/Table_S6.pdf]

Table S6. Targets and central nodes (in bold the ones shared by all species; underlined the ones shared by Donkey and Goat).

**Species**	**Targets**	**Central nodes**
Cow	YOD1 MFSD8 MDM4	**MDM5**<br>YOD1<br>MFSD8
Donkey	ZNF264 MDM4 IKZF3<br>ZNF644 ZNF587 ZBTB37<br>YOD1 SOCS7 RBM12B<br>PRORP LCOR FAM104A<br>CSNK2A1 ADIPOR2<br>CEP120	CSNK2A1<br>IKZF3<br>ZNF587<br>CEP120<br>ZNF644<br>LCOR<br>**MDM4**<br>SOCS4
Goat	YOD1 ZNF264 MDM4<br>AGO-01 ZBTB37 SOCS7<br>MFSD8 KPNA6 CEBPG<br>BZW1 ATXN1 ARIH1<br>KMT2D EPHA4	ATXN1<br>KPNA6<br>ARIH1<br>KMT2D<br>**MDM4**<br>SOCS4<br>ZNF264<br>EPHA4
