# Supplementary material for: Transcriptomic Characterization of Cow, Donkey and Goat Milk Extracellular Vesicles Reveals Their Anti-inflammatory and Immunomodulatory Potential
Source: Int J Mol Sci. 2021 Nov 25;22(23):12759. doi: 10.3390/ijms222312759 (PMC8657891; doi:10.3390/ijms222312759)
Supplement: Supplementary file 1 [file ijms-22-12759-s001.zip › Supplementary_files/Figure_S1.pdf]

Udder from SRA archive and mEV gene set comparison.

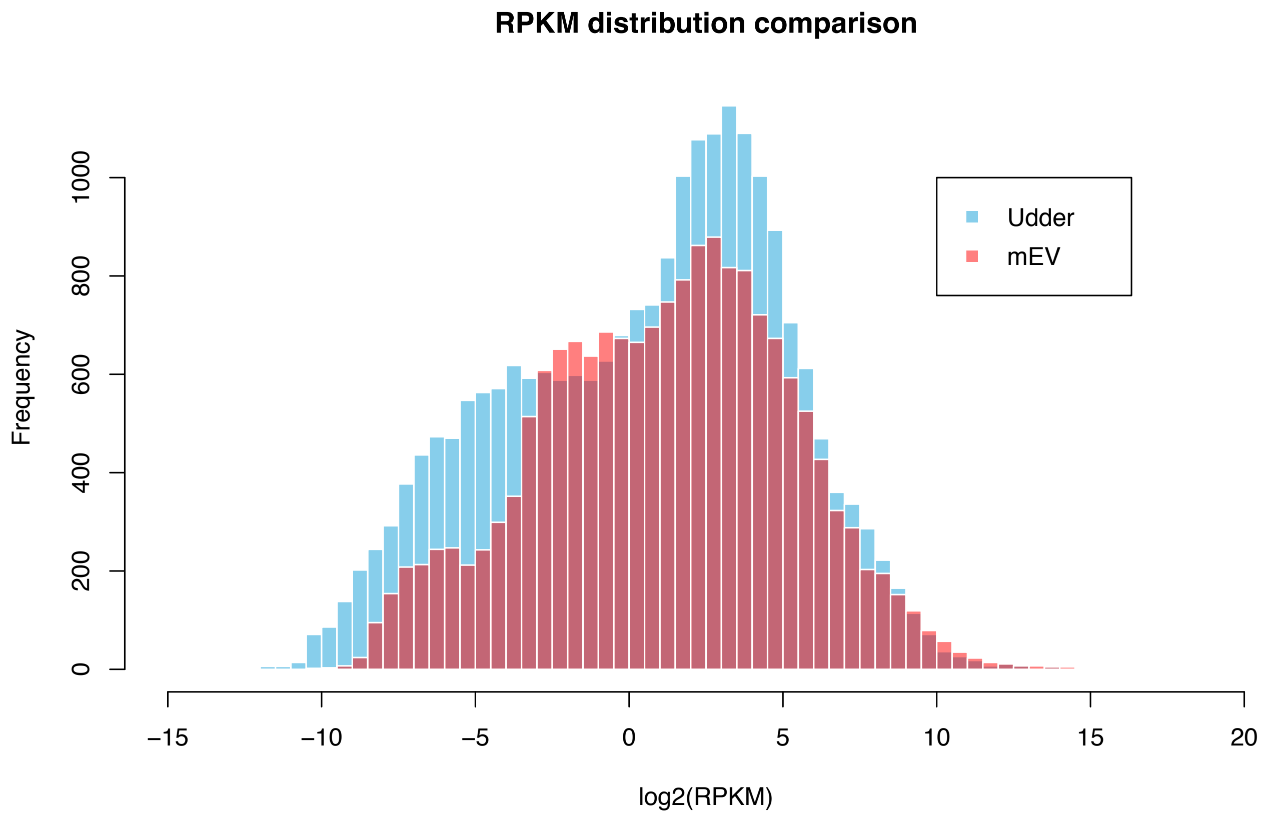

Figure S1. Histogram of RPKM mean for udder data (blue) downloaded from SRA database (BioSamples: SAMN12831050, SAMN12831049, SAMN14600526 and SAMN14600525) and mEV gene set (red).
