# Supplementary material for: Transcriptomic Characterization of Cow, Donkey and Goat Milk Extracellular Vesicles Reveals Their Anti-inflammatory and Immunomodulatory Potential
Source: Int J Mol Sci. 2021 Nov 25;22(23):12759. doi: 10.3390/ijms222312759 (PMC8657891; doi:10.3390/ijms222312759)
Supplement: Supplementary file 1 [file ijms-22-12759-s001.zip › Supplementary_files/Figure_S4.pdf]

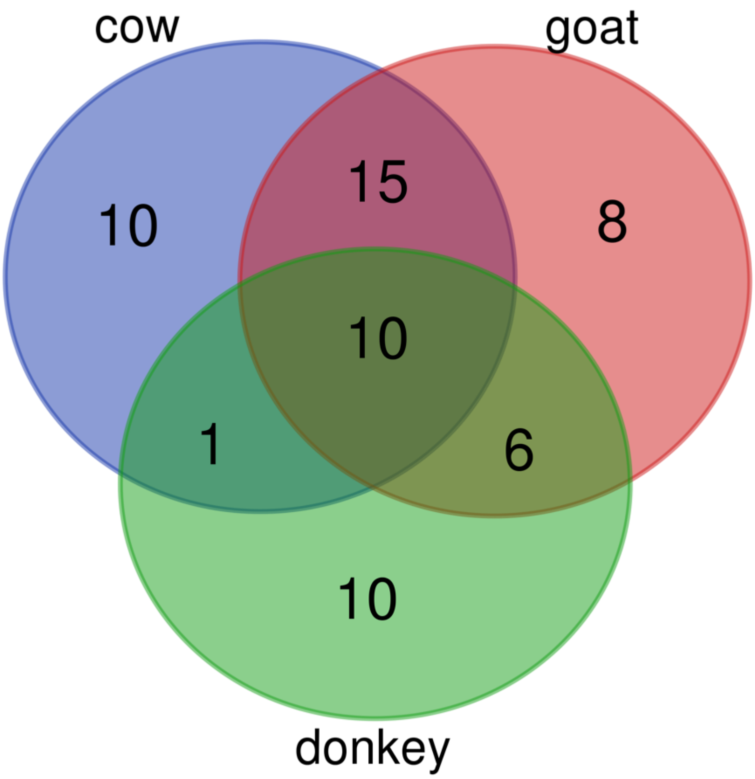

Figure S4. Venn Diagram representing consistencies of shared miRNAs among the highly expressed (covered by 95% RPKM) in mEVs of the three species.
