# Supplementary figures and images for: Transcriptomic Characterization of Cow, Donkey and Goat Milk Extracellular Vesicles Reveals Their Anti-inflammatory and Immunomodulatory Potential
Source: Int J Mol Sci. 2021 Nov 25;22(23):12759. doi: 10.3390/ijms222312759 (PMC8657891; doi:10.3390/ijms222312759)

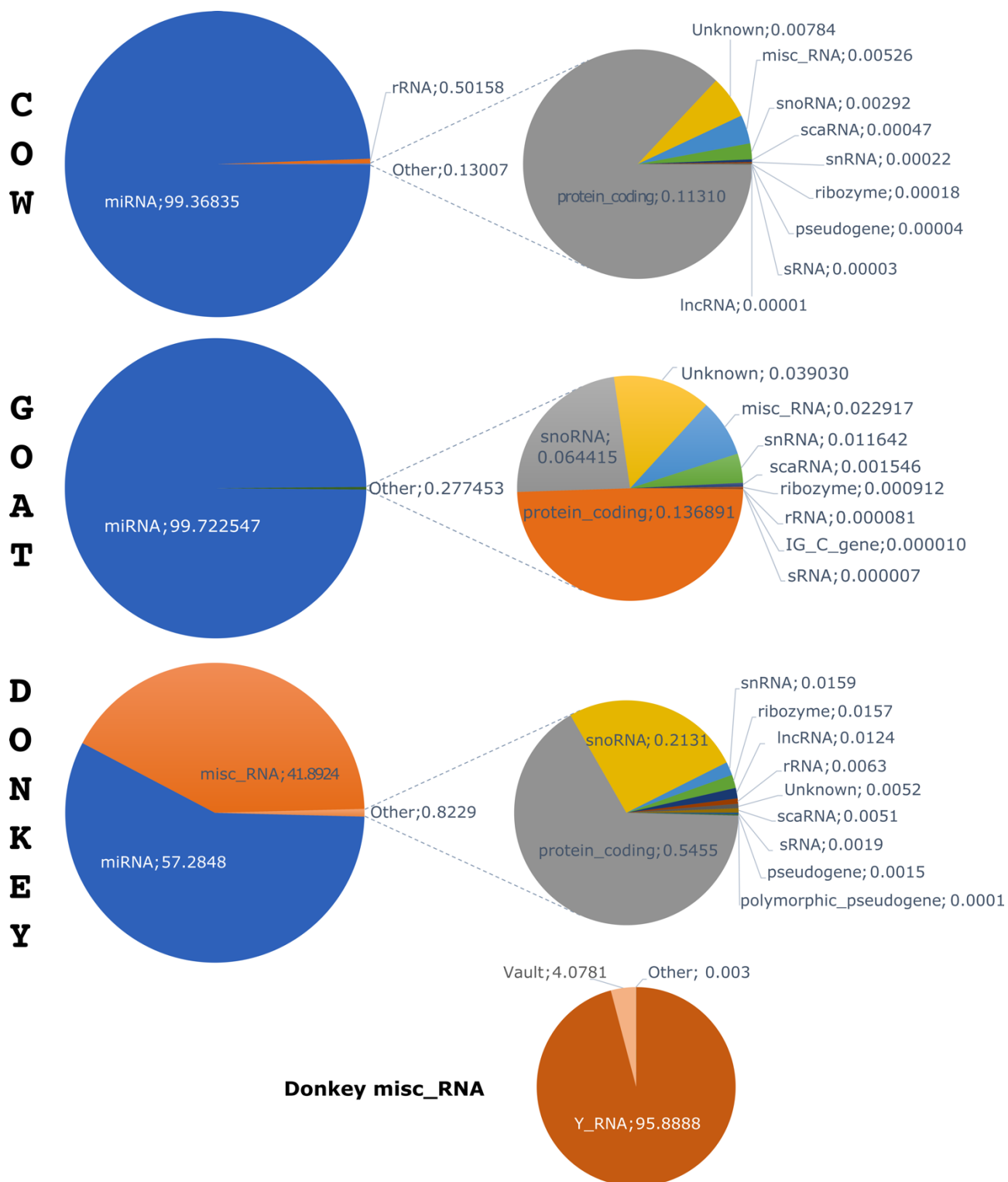

**Figure S3.** Small RNA features in cow, goat and donkey mEVs.

Supplement: Supplementary file 1 [file ijms-22-12759-s001.zip › Supplementary_files/Figure_S3.pdf]

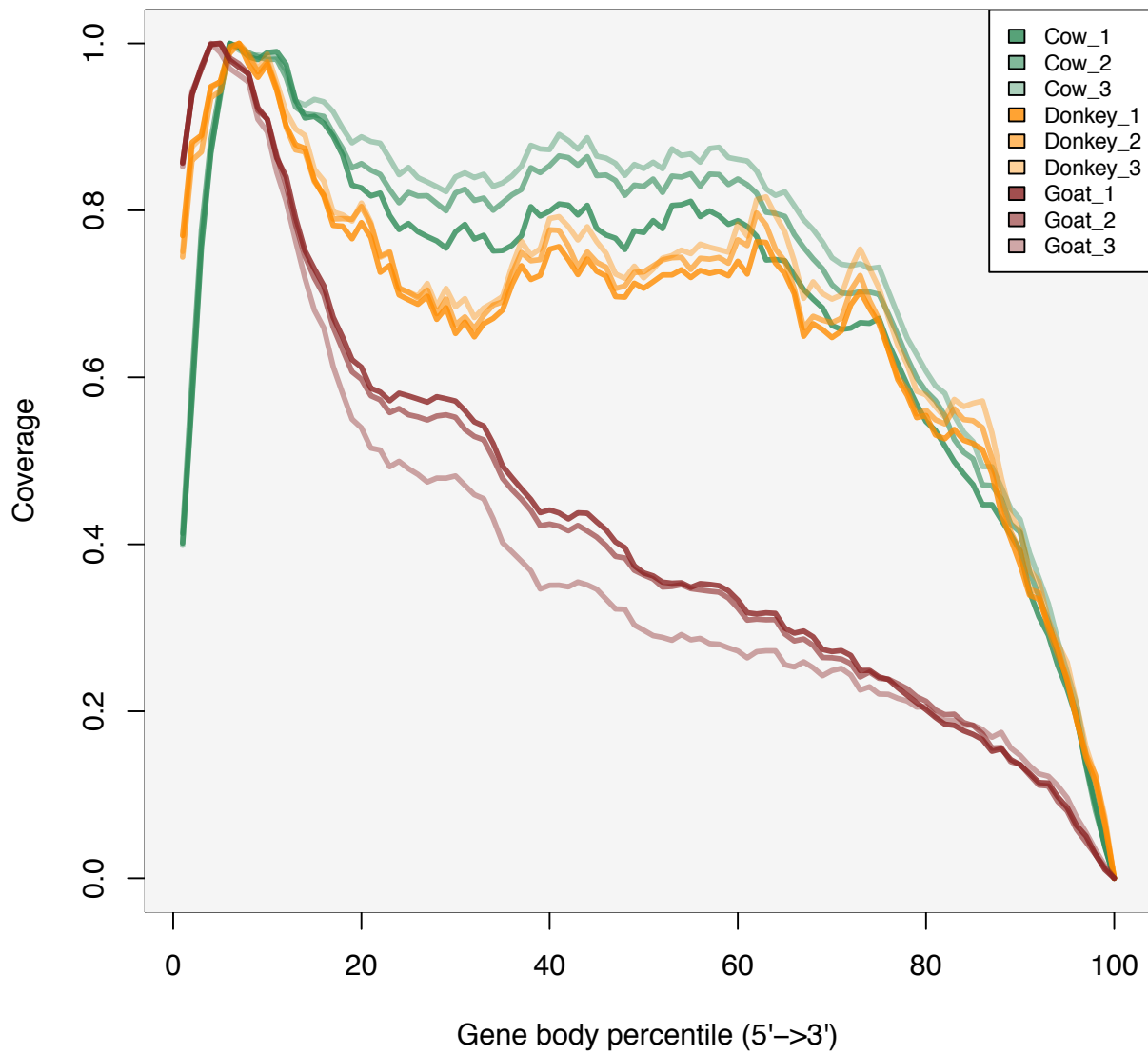

**Figure S2.** Gene body coverage for mRNA mEV cargo of the three species.

Supplement: Supplementary file 1 [file ijms-22-12759-s001.zip › Supplementary_files/Figure_S2.pdf]
